# The Influence of the Big Five Personality Factors on Mental Health Before and During the COVID-19 Pandemic: A Prospective Study

**DOI:** 10.1177/00332941241300949

**Published:** 2024-11-21

**Authors:** Peter G. van der Velden, Lutz Wittmann, Carlo Contino, Erik van der Meulen, Marcel Das, Hendri Adriaens

**Affiliations:** Tranzo, 7899 Tilburg University, Tilburg, The Netherlands; 192233 Centerdata, Tilburg, The Netherlands; 191625 International Psychoanalytic University Berlin, Berlin, Germany; 634366 Fonds Slachtofferhulp, The Hague, The Netherlands; Academy of Healthcare, 84808 NHL Stenden University of Applied Sciences, Leeuwarden, The Netherlands; Department of Research, Education and Internationalisation, NHL Stenden University of Applied Sciences, Leeuwarden, The Netherlands; 192233 Centerdata, Tilburg, The Netherlands; Tilburg School of Economics and Management, 7899 Tilburg University, Tilburg, The Netherlands; 192233 Centerdata, Tilburg, The Netherlands

**Keywords:** Big five personality factors, anxiety, depression, COVID-19, gender

## Abstract

The Big Five personality factors (PF) are considered to be predictive of mental health problems, but it is unclear if these factors equally contributed to mental health problems during the COVID-19 pandemic compared to before the pandemic. This prospective study aimed to fill this knowledge gap. For this purpose data was extracted from the population-based LISS-panel. We included adult respondents (N^males^ = 1,838, N^females^ = 1892) who participated in three surveys before the pandemic (T1^March-2019^, T2^May-2019^, T3^November-2019^) and in three surveys during the pandemic in 2020 (T4^March-2020^, T5^May-2020^, T6^november-2020^). Multivariate logistic regression analyses were used to examine and compare the longitudinal associations between PF at T2^May-2019^ and moderate-severe anxiety and depressions symptoms (ADS) at T3^November-2019^, and longitudinal associations between PF at T5^May-2020^ and ADS at T5^November-2020^ among males and females. Control variables (pre-existing ADS, lack support, demographics) were retrieved from the T1^March-2019^ and T4^March-2020^ surveys, respectively. For the present study we distinguished five levels (very high to very low) of each PF. For both sexes, those with (very) low emotional stability and/or conscientiousness had considerably higher rates of ADS compared to those with very high levels of the same trait. These findings were similar both before and during the pandemic. Moreover, we found no indications that those with a certain level of a PF during the pandemic were more of less at risk for ADS or persistent ADS, than those with the same level of the same PF before the pandemic. Thus, we found no indications that the pandemic affected the impact of personality factors on moderate-severe anxiety and depressions symptoms.

## Introduction

The Big Five personality factors extraversion, agreeableness, openness, conscientiousness, and especially emotional stability (neuroticism) are associated with a wide range of mental health issues such as depression, fatigue, burnout, eating disorders, subjective well-being, resilience and mental health treatment outcomes (Alarcon et al., 2009; Anglim et al., 2020; Bucher et al., 2019; Farstad et al., 2016; Hakulinen et al., 2015; Jourdy & Petot, 2017; Khazanov & Ruscio, 2016; Klein et al., 2011; Kotov et al., 2010; Oshio et al., 2018; Stephan et al., 2020; Strickhouser et al., 2017).

The disruptions and stress people faced due to the COVID-19 pandemic raise the question to what extent personality factors contributed to or protected against mental health problems during this pandemic (cf. Robinson et al., 2022). As noted by Rettew et al. (2021), it is important not to assume that personality traits are universally adaptive for all types of stressful circumstances such as this pandemic. To date, several cross-sectional studies assessed the associations between the Big Five personality factors and mental health problems such as anxiety and depression symptomatology during the pandemic (cf. Mansueto et al., 2022; Pereira et al., 2022; Pradhan et al., 2022; Shokrkon et al., 2021; Wang et al., 2022; Yu & Hu, 2022; Zager Kocjana et al., 2021).

However, cross-sectional studies do not clarify the extent to which personality factors predict mental health problems during the pandemic *over and above* pre-existing mental health problems, while research has abundantly shown that mental health problems are strongly predicted by past mental health problems (cf. Velden et al., 2023). Not controlling for pre-existing mental health problems therefore increases the risk for overestimating the effects of personality on mental health problems (cf. Hakulinen et al., 2015; Khazanov & Ruscio, 2016). To the best of our knowledge, only the study by Anglim and Horwood (2021) examined the extent to which the predictive values of personality during the pandemic differs from the influence before the outbreak. They assessed the extent to which the pandemic moderated the relationships between the Big Five personality factors and (among others) subjective wellbeing (SWB) and negative affect (NA), using the cross-sectional data of one pre-COVID-19 (July 2017) and one similar recruited post-outbreak sample (July 2020). Results showed that the relationships were relatively stable, but because analyses were not controlled for pre-existing SWB and NA it cannot be ruled out that changes in relationships were nonetheless present (besides that pre-COVID NA items referred to the past weeks and the post-outbreak items referred to the past months).

### Prospective COVID-19 Studies on Role of Big Five

A limited number of studies on predictive values of the Big Five personality factors of mental health used a prospective study design with pre-outbreak mental health assessments among the same adult study sample. Proto and Zhang (2021) and Staneva et al. (2022) extracted data from the large population-based UK Household Longitudinal Survey, and analyzed the effects of the Big Five personality factors assessed in 2011–2013. Psychological distress (GHQ-12) assessed in 2017–2019 was used as the pre-COVID-19 distress measure in both studies. Proto and Zhang (2021) examined mental health changes until January 2021 (including April, May, July, September, and November 2020) and found that pre-outbreak extraversion and openness were associated with higher mental health deterioration while lower pre-outbreak agreeableness were associated with less mental health deterioration. Neuroticism was strongly associated with mental health problems cross-sectionally, but did not lead to stronger deterioration. Staneva et al. (2022) focused on changes in distress among six age categories of females and males during the first months after the outbreak (April, May, and June 2020), among subgroups with low and high scores on the Big Five separately. They found that in general an increase in psychological distress symptoms was more pronounced among subgroups with higher neuroticism, extroversion, and openness scores than in subgroups with lower scores. Among females each subgroup of personality factors (low and high) was predictive of an increase in psychological distress in all age categories, and especially youngsters, with one exception for those with low agreeableness levels. Among males, an almost similar pattern was observed with a few more exceptions especially for those with low neuroticism and extraversion levels.

The two-wave study by Muro et al. (2021) focused on respondents recruited via social networks through Google Forms following virtual snowball sampling. Results showed that neuroticism at baseline (March 2020) predicted depression and anxiety caseness 30–45 days after the Spanish official confinement period, over and above, among other factors, baseline depression and anxiety scores, respectively. Rettew et al. (2021) assessed changes in daily mood and stress ratings from pre-COVID-19 (January 2020 to outbreak) to COVID-19 (outbreak to May 2020) among a convenience sample of first-year students. Significant interaction effects, which were controlled for potential confounders, demonstrated that the decrease of mood during COVID-19 was less pronounced for those with higher levels of neuroticism and lower levels of extraversion, openness, and agreeableness. However, with one exception (extraversion), the effect sizes were modest. The study by Szenczy and Nelson (2021) among undergraduate students found that pre-pandemic neuroticism, assessed between May 2018 and February 2020, significantly predicted changes in distress and positive mood during the first months after the outbreak (April-May), but not changes of fear/obsessions, when controlled for potential confounders. However, the effect sizes were small (partial correlations were .15 and −.16, respectively). Kekäläinen et al. (2021) examined a population-based cohort study of 51–59 years-old Finnish women. Findings showed that pre-pandemic higher extraversion levels and lower neuroticism levels assessed between November 2018 and March 16, 2020, were associated with less depression symptoms, but not with the change in depression symptoms. The study by Rius Ottenheim et al. (2022) among adults (18–65 years), based on data of three prospective cohorts (NESDA, NESDO, and NOCDA), showed that of the Big Five personality factors assessed in 2007–2009 (standardized scores of two different Big Five measures), only neuroticism independently predicted worsening of anxiety and depression symptoms during the first months post-outbreak compared to before the outbreak (means of symptom scores up to four most recent available waves, collected between 2006 and 2016). However, the strength of the predictive value of neuroticism is unclear.

Importantly, none of these studies also examined these factors during the pandemic. Although personality factors are considered relatively stable traits they may change over time (Bleidorn et al., 2018; Debast et al., 2014; Karsten et al., 2012) such as due to experiences during the pandemic (Specht et al., 2011; Sutin et al., 2020). If the predictive values of pre-outbreak personality factors for mental health problems would for instance decrease over time, it would therefore remains unclear if the role of personality factors decreased due longer time intervals or if the role personality factors decreased due to the pandemic.

### Research Questions Current Study

In sum, there is a lack of prospective studies among the general population based on probability samples that compared the predictive values of the Big Five personality factors for mental health problems *before* with the predictive values of these factors assessed *during* the pandemic for mental health problems during the pandemic, that take pre-existing mental health problems into account to unravel the impact of personality factors on post-outbreak mental health problems. In addition, there is a lack of studies that controlled for social support while this pandemic -due to all lockdowns-impacted social interactions which may have increased the lack of social support (cf. Birkeland et al., 2017; Cohen & Wills, 1985; Guan et al., 2022; Koenen et al., 2017; Steel et al., 2014).

To fill this gap, the current 6-wave prospective population-based study was conducted in which we assessed and compared the prospective associations between personality factors (T5) and anxiety and depression symptoms (ADS) *during the pandemic* (T6) with the prospective associations between personality factors (T2) and ADS *before the pandemic* (T3) with a similar a time-interval (six months between personality and ADS assessments). In other words, are the prospective associations between the Big Five personality factors and ADS influenced by the COVID-19 pandemic? Current longitudinal studies (see above) especially examined the extent to which personality factors are associated with worsening mental health problems. A related but unanswered relevant question is to what extent personality factors are associated with persistent mental health problems among those with pre-existing mental health problems (T1 and T3 respectively). Are they associated with the recovery of mental health problems? The main research question was:

To what extent does the prevalence of (persistent) moderate-severe ADS at T6 among adult with very high, high, medium, low and very low levels of the Big Five personality factors during the pandemic (assessed at T5) differ from the prevalence of (persistent) moderate-severe ADS at T3 among their counterparts with similar levels of the same personality factors before the pandemic (assessed at T2) while controlling for relevant confounders (assessed at T4 and T1 respectively).

To answer this main research question, we first examined: A.) the extent to which the prevalence of (persistent) moderate-severe anxiety and depression symptoms (ADS) among adults with high, medium, low and very low levels of the Big Five personality factors differ from the prevalence of moderate-severe ADS among adults with very high levels of the Big Five personality, *before the COVID-19 pandemic* [Big Five assessed in April-May 2019 (T2), ADS assessed in November-December 2019 (T3)], while controlling for relevant confounders [assessed in March 2019 (T1)]; and B.) the extent to which the prevalence of (persistent) moderate-severe ADS among adults with high, medium, low and very low levels of the Big Five personality factors differ from the prevalence among adults with very high levels of the Big Five personality, *during the COVID-19 pandemic* [Big Five assessed in April-May 2020 (T5), ADS assessed in November-December 2020 (T6)] while controlling for relevant confounders [assessed in March 2020 (T4)].

Given the differences between males and females on the Big Five personality factors (Costa et al., 2001; Feingold, 1994; Schmitt et al., 2008) and in line with Staneva et al. (2022) we examined the longitudinal associations among males and females separately.

### COVID-19 and the Netherlands

In the Netherlands, the COVID-19 outbreak occurred in March 2020. At the end of March 9762 COVID-19 patients were registered, of whom 639 had died. Several periods of (partial) lockdowns of, for instance, schools, cinemas, and cafes followed until the end of 2021 (for details, see interactive COVID-19 Government Response Tracker; Oxford University, 2022). Widespread financial aid programs were launched to aid small and big business owners to retain their personnel and prevent a lockdown-related economic backlash. The vaccination program started in January 2021, giving room to roll back some of the lockdown measures and alleviate the disease burden of the pandemic.

## Method

### Participants and Procedures

For the present study, data were extracted from six surveys conducted with the *Longitudinal Internet studies for the Social Sciences* (LISS) panel (Scherpenzeel & Das, 2011). This panel is based on a traditional probability sample drawn from the Dutch population register by Statistics Netherlands and administered by Centerdata. Respondents cannot sign themselves up as respondents for the panel. The initial set-up was funded by the Dutch Research Council (NWO). Members who do not have a computer and/or internet access are provided with the necessary equipment at home. Respondents receive an incentive of 15 euros per hour of interview time. In accordance with the General Data Protection Regulation (GDPR), participants gave explicit digital consent for the use of the collected data for scientific and policy relevant research. For further information about the LISS panel, all conducted studies since 2007, and open access data see: https://www.dataarchive.lissdata.nl (in English). Data are available to any interested researcher through the LISS Data Archive, free of charge. The archive received the CoreTrustSeal certification, based on the World Data System (WDS) of the International Science Council and the Data Seal of Approval (DSA) catalogue and procedures.

Table 1 provides an overview of the six surveys and response rates. For all surveys, reminders are sent during the month of data collection. Those who did not respond, were re-invited one month later. The VICTIMS survey was approved by an Internal Review Board of Centerdata, consisting of independent, internal and external reviewers. These reviewers were not involved in the development of the VICTIMS-study. The Health and Personality survey was evaluated and approved by an independent Board of Overseers, an IRB of Centerdata until 2014. Since our research did not impose a certain behavior, our research did not need the approval of a Dutch Medical Ethical Testing committee according to Dutch Law. The authors assert that all procedures contributing to this work comply with the ethical standards of the relevant national and institutional committees on human experimentation and with the Helsinki Declaration of 1975, as revised in 2008.

**Table 1.** Overview Six Surveys Conducted With the LISS Panel Used in Present Study.

Time	Survey	Invited	Response (%)
T1 March 2019	Longitudinal VICTIMS study	6298	83.2
T2 May 2019	Longitudinal core study personality	6218	80.7
T3 November 2019	Longitudinal core study health	5954	86.4
T4 March 2020	Longitudinal VICTIMS study	6568	83.6
T5 May 2020	Longitudinal core study personality	6969	84.1
T6 November 2020	Longitudinal core study health	6832	83.6

In total, 3730 respondents participated in all six surveys. To optimize the representativeness of the study sample, the data were weighted for Dutch adult population (2019). Based on the (open access) data of Statistics Netherlands with respect to age (14 categories), gender (two categories), and marital status (two categories), we composed 56 exclusive demographic profiles of the Dutch adult population. All results are based on the weighted data.

## Measures

### Demographics

Demographics such as sex, age, education, employment status, and marital status were assessed in March 2019 (T1) and March 2020 (T4).

### Personality Factors

The Big Five personality factors, extraversion, agreeableness, conscientiousness, neuroticism (emotional stability), and openness, were examined in May 2019 (T2) and May 2020 (T5), with the 50-item International Personality Item Pool (IPIP; Goldberg, 1992). Each of the five scales consist of 10 items (scores ranges from 10 to 50). Individual items are statements describing an individual’s characteristics and items are scored on a 5-point Likert scale with scores ranging from 1 (very inaccurate) to 5 (very accurate). After reverse coding, cores are summed with greater scores indicating higher levels of extraversion, agreeableness, conscientiousness, emotional stability, and openness, respectively (Cronbachs Alpha’s (CAs) for males and females at T2 and T5: emotional stability: .888 ≤ CAs ≤.892; agreeableness: .802 ≤ CAs ≤.844; extraversion: .881 ≤ CAs ≤.897; conscientiousness: .770 ≤ CAs ≤.798; openness: .756 ≤ CAs ≤.781).

### Anxiety and Depression Symptoms

Anxiety and depression symptoms (ADS) in March 2019 (T1) and November 2019 (T3) and in March 2019 (T4) and November 2020 (T6) were examined using the 5-item Mental Health Index (MHI-5: Means-Christensen et al., 2005; Ware & Sherbourne, 1992). The MHI-5 asks respondents to rate their mental health during the past month on 6-point Likert scales (0 = never to 5 = continuously). After recoding the three negatively formulated items, the total scores were computed and multiplied by four (to arrive at a 0–100 scale; CAs for males and females at T1, T3, T4 and T6: .852 ≤ CAs ≤.878). We used the cut-off score of ≤ 60 (Perenboom et al., 2000) to calculate the prevalence of moderate-severe ADS symptom levels.

### Lack of Emotional Support

Lack of emotional support in response to problems was examined in March 2019 (T1) and March 2020 (T4) by the 8-item subscale Lack of emotional support of the Social Support List-Discrepancy (SSL-D; Bridges et al., 2002; Sonderen, 2012). The SSL-D items apply 4-point Likert scales (1 = I miss this, I would like it to happen more often to 4 = It happens too often). For the present study, total scores were subtracted from the total maximum scores whereby higher scores reflect more lack of emotional support (CAs for males and females at T1 and T4 .877 ≤ CAs ≤.892).

### Statistical Analyses

To answer the first two research questions we applied Multivariate Logistic Regression Analyses (MLRA) with moderate-severe ADS as the dependent variable. In line with Staneva et al. (2022), the scores of each personality factor as assessed in April 2019 (T2) were divided into five exclusive categories (cf. Aben et al., 2020; Besser et al., 2007; Kekäläinen et al., 2021; Klein et al., 2011; Strohmaier et al., 2024). The five categories (very low, low, medium, high and very high scores) each covered about 20% of the scores of the respective factor (Emotional stability: very low = low thru 28, low = 29 thru 32, medium = 33 thru 36, high = 37 thru 40, very high = 41 thru hi. Agreeableness: very low = low thru 33, low = 34 thru 37, medium = 38 thru 39, high = 40 thru 42, very high = 43 thru hi. Extraversion: very low = low thru 26, low = 27 thru 30, medium = 31 thru 33, high = 34 thru 37, very high = 38 thru hi. Conscientiousness: very low = low thru 32, low = 33 thru 35, medium = 36 thru 39, high = 40 thru 41, very high = 42 thru hi. Openness: very low = low thru 30, low = 31 thru 33, medium = 34 thru 36, high = 37 thru 39, very high = 40 thru hi). The cut-off scores of the categories of the personality factors were applied to the factors assessed in April 2020 (T5). The mean scores of categories of a personality assessed in 2019 and 2020 were similar among males and among females (see Appendix 1). The differences in scores of the categories across the five factors were meaningful. For example the Cohens D’s of very high versus high scores across the five factors were all large (≥2.4) and the Cohens D’s of very high versus very low scores were all very large (≥5.5) indicating (very) strong differences in scores.

In the MLRA, a specific personality factor (assessed in May) was entered as predictor while controlling for relevant confounders such as demographics, pre-existing lack of emotional support and pre-existing ADS assessed in March in the same year. Persistent ADS was defined as having moderate-severe ADS in March and November of the same year. Thus, the analyses with persistent ADS as dependent variable were conducted among those with pre-existing ADS (assessed in March of each year).

To answer the main research question, the prevalence of (persistent) ADS during the pandemic (T6) among those with very high, high, medium, low and very low levels of each personality factor (T5) should be compared with the prevalence of (persistent) ADS before the pandemic (T3) among those with the same levels of the same personality factor (T2). For instance, the prevalence of (persistent) ADS at T6 among males with very low levels of emotional stability at T5 should be compared with the prevalence of (persistent) ADS at T3 among males with very low levels of emotional stability at T2. However, as could be expected, there is a considerable overlap between subgroups with the same levels of a personality factor at T5 and at T2 indicating that these subgroups are not independent and thus cannot be compared directly (nor can the adjusted Odds Ratios). To solve this non-independence problem we performed a stratified random sampling among males to obtain independent comparable subgroups of males and a performed a similar stratified random sampling among females (this sampling was performed separately for each personality factor level).

We finally cross-wise examined the differences in the prevalence of (persistent) ADS between these independent subgroups as follows (we use low emotional stability and ADS as an example). 1.) we compared the prevalence of ADS of subgroup A^low emotional stability^ at T3 with the prevalence of ADS of subgroup B^low emotional stability^ at T6, and 2.) we compared the prevalence of ADS of subgroup B^low emotional stability^ at T3 with the prevalence of ADS of subgroup A^low emotional stability^ at T6. The differences were examined using MLRA with age, marital status, primary occupation, education level, lack of emotional support and ADS examined in March in the same year as control variables. Similar analyses were conducted for low, medium, high and very high scores of emotional stability.

This analytic strategy was applied to the other four personality factors and all analyses were repeated among female respondents. Given the number of tests, for each research question (Table) we applied the Benjamini-Hochberg correction of *p*-values (Benjamini & Hochberg, 1995).

## Results

### Characteristics Study Sample

In Table 2, the characteristics of the study sample are presented.

**Table 2.** Characteristics Study Sample (*N* = 3730).

	Males		Females	
	March 2019 (T1)	March 2020 (T4)	March 2019 (T1)	March 2020 (T4)
	*N (%)*	*N (%)*	*N (%)*	*N (%)*
Marital status				
- Married	899 (48.9)	908 (49.4)	897 (47.4)	904 (47.8)
- Unmarried	939 (51.1)	930 (50.6)	995 (52.6)	988 (52.2)
Primary occupation				
- Unemployed	743 (40.4)	734 (39.9)	989 (52.3)	973 (51.5)
- Employed	1095 (59.6)	1104 (60.1)	903 (47.7)	918 (48.5)
Education level				
- Low	419 (22.8)	390 (21.2)	520 (27.5)	511 (27.0)
- Medium	675 (36.7)	683 (37.1)	698 (36.9)	675 (35.7)
- High	744 (40.5)	765 (41.6)	674 (35.6)	706 (37.3)
Background				
- Dutch/western	1670 (90.9)	Idem	1745 (92.2)	Idem
- Non-western	168 (9.1)	Idem	147 (7.8)	Idem

	*M* (SD)	*M* (SD)	*M* (SD)	*M* (SD)
Age	48.6 (18.3)	49.7 (18.3)	50.1 (19.0)	51.2 (19.0)
Anxiety and depression symptoms	75.5 (16.5)	75.4 (16.4)	72.6 (17.0)	72.4 (16.4)
Lack of emotional support	10.3 (3.5)	10.0 (3.2)	10.5 (3.7)	10.3 (3.5)

All results are based on the weighted sample and due to the weighting numbers (*N*) may differ slightly.

### Prevalence of ADS Among Males

Of all males, 19.6% and 19.4% had moderate-severe ADS before (T3) and during the pandemic (T6), respectively. In Table 3 the results of the MLRA among males before and after the Benjamini-Hochberg correction are presented. They show that before as well as during the pandemic males with medium, low and very low levels of emotional stability, more often suffered from moderate-severe ADS about six months later than males with very high levels of emotional stability. After the Benjamini-Hochberg correction both before and during the pandemic males with very low levels of conscientiousness significantly more often suffered from ADS than males with very high conscientiousness levels. In contrast to during the pandemic, before the pandemic males with low and very low levels of extraversion had a higher prevalence of moderate-severe ADS than males with very high levels of extraversion. A similar pattern was found for very low levels of agreeableness before and during the pandemic. With respect to openness, males who did not have very high levels of openness did not differ in the prevalence of moderate-severe ADS from males with very high levels of openness: not before and not during the pandemic after the Benjamini-Hochberg correction.

**Table 3.** Prospective Associations Between Personality Factors and Prevalence of ADS Among Males (*N* = 1838).

	Moderate-Severe Anxiety and Depression Symptoms (ADS)					
	November 2019 (T3)			November 2020 (T6)		
	*N* ^*total*^	*n (%)*	*aOR (95%CI)* a	*N* ^*total*^	*n (%)*	*aOR (95%CI)* a
Emotional stability						
- Very high^b^	467	8 (1.7)	1	523	12 (2.3)	1
- High	478	23 (4.8)	1.58 (0.68–3.68)	449	22 (4.9)	1.45 (0.68–3.09)
- Medium	327	49 (15.0)	4.06 (1.83–9.01)^***^	320	49 (15.3)	3.13 (1.55–6.34)^**^
- Low	293	118 (40.3)	9.58 (4.40–20.87)^***^	298	122 (40.9)	7.41 (3.76–14.60)^***^
- Very low	272	163 (59.9)	11.11 (5.00–24.71)^***^	248	151 (60.9)	12.66 (6.32–25.33)^***^
Agreeableness						
- Very high^b^	212	40 (18.9)	1	182	35 (19.2)	1
- High	315	34 (10.8)	0.87 (0.45–1.70)	314	38 (12.1)	0.51 (0.27–0.97)^*c^
- Medium	343	59 (17.2)	1.44 (0.78–2.66)	335	47 (14.0)	0.65 (0.36–1.19)
- Low	468	85 (18.2)	1.46 (0.82–2.61)	478	80 (16.7)	0.77 (0.44–1.35)
- Very low	501	143 (28.5)	2.11 (1.19–3.73)^*^	528	155 (29.4)	1.07 (0.62–1.84)
Extraversion						
- Very high^b^	392	35 (8.9)	1	366	43 (11.7)	1
- High	347	56 (16.1)	1.60 (0.93–2.76)	351	43 (12.3)	0.89 (0.51–1.55)
- Medium	283	60 (21.2)	1.59 (0.92–2.75)	324	63 (19.4)	1.63 (0.97–2.76)
- Low	397	90 (22.7)	1.98 (1.19–3.29)^**^	360	83 (23.1)	1.65 (0.99–2.73)
- Very low	419	120 (28.6)	1.96 (1.18–3.25)^**^	436	123 (28.2)	1.57 (0.97–2.54)
Conscientiousness						
- Very high^b^	357	38 (10.6)	1	393	37 (9.4)	1
- High	244	29 (11.9)	0.71 (0.37–1.35)	237	31 (13.1)	1.18 (0.63–2.21)
- Medium	524	77 (14.7)	0.87 (0.53–1.45)	497	78 (15.7)	1.53 (0.92–2.56)
- Low	330	58 (17.6)	1.09 (0.64–1.86)	316	61 (19.3)	1.78 (1.04–3.04)^*^^c^
- Very low	384	159 (41.4)	2.47 (1.52–4.02)^***^	395	149 (37.7)	2.24 (1.36–3.70)^**^
Openness						
- Very high^b^	399	70 (17.5)	1	389	58 (14.9)	10
- High	363	43 (11.8)	0.55 (0.32–0.94)^*^^c^	349	65 (18.6)	1.21 (0.74–1.98)
- Medium	407	76 (18.7)	1.44 (0.90–2.30)	431	64 (14.8)	0.90 (0.55–1.48)
- Low	357	81 (22.7)	1.16 (0.71–1.89)	344	84 (24.4)	1.42 (0.87–2.33)
- Very low	312	91 (29.2)	1.49 (0.90–2.46)	325	85 (26.2)	1.25 (0.76–2.07)

aOR = Odds ratio adjusted for age, education level, marital status, primary occupation, ethnicity, anxiety and depression symptoms and lack of emotional support assessed in March of the same year (T1 or T4). 95% CI = 95% confidence interval. All results are based on the weighted sample and due to the weighting numbers (*N*, *n*) may differ slightly.

^a^*p*-values before the Benjamini-Hochberg correction (^*^p < .05, ^**^p < .01, ^***^p < .001).

^b^Reference category. Personality factors were examined in May of the same year (T2 or T5).

^c^Not significant after the Benjamini-Hochberg correction.

The cross-wise MLRA showed no significant differences in the prevalence of moderate-severe ADS between males with very high, high, medium, low and very low levels of emotional stability, extraversion, agreeableness, extraversion and openness before the pandemic compared to males with the same levels of the same personality factors during the pandemic. Before the correction only one out of the 50 comparisons was significant indicating a higher prevalence of ADS among males with high openness levels during the pandemic compared to prevalence of ADS among males with high openness levels before the pandemic. However, after this correction this difference was no longer significant (see Appendix 2).

### Prevalence of ADS Among Females

Of all females, 24.0% and 23.8% had moderate-severe ADS at T3 and T6 respectively. The results of the MLRA among females are presented in Table 4. With respect to emotional stability an almost identical pattern was observed as among males. But in contrast to males, females with medium levels of emotional stability before and during the pandemic did not have a higher prevalence of ADS six months later than females with a very high level of emotional stability, after the Benjamini-Hochberg correction. Before the pandemic females with low conscientiousness were more at risk for ADS, while during the pandemic females with very low levels of conscientiousness were more at risk for ADS compared to their counterparts with very high levels of conscientiousness after the Benjamini-Hochberg correction. No differences in the prevalence of moderate-severe ADS were observed between females with very high levels of agreeableness, extraversion and openness versus females with high, medium, low and very low levels before and during the pandemic, after the Benjamini-Hochberg correction.

**Table 4.** Prospective Associations Between Personality Factors and Prevalence of ADS Among Females (*N* = 1892).

	Moderate-Severe Anxiety and Depression Symptoms (ADS)					
	November 2019 (T3)			November 2020 (T5)		
	*N* ^*total*^	*n (%)*	*aOR (95%CI)* a	*N* ^*total*^	*n (%)*	*aOR (95%CI)* a
Emotional stability						
- Very high^b^	308	15 (4.9)	1	332	13 (3.9)	1
- High	403	25 (6.2)	0.94 (0.47–1.88)	385	29 (7.5)	1.37 (0.67–2.77)
- Medium	378	51 (13.5)	1.44 (0.76–2.71)	377	58 (15.4)	2.40 (1.23–4.65)^**^^c^
- Low	369	118 (32.0)	2.39 (1.29–4.41)^**^	362	102 (28.2)	3.13 (1.63–6.00)^***^
- Very low	433	244 (56.4)	3.89 (2.11–7.20)^***^	438	249 (56.8)	6.69 (3.49–12.80)^***^
Agreeableness						
- Very high^b^	528	124 (23.5)	1	541	111 (20.5)	1
- High	466	93 (20.0)	0.67 (0.45–0.98)^*^^c^	489	114 (23.3)	1.03 (0.72–1.48)
- Medium	347	71 (20.5)	0.74 (0.49–1.12)	325	70 (21.5)	0.97 (0.64–1.45)
- Low	356	106 (29.8)	1.11 (0.75–1.66)	335	98 (29.3)	1.13 (0.77–1.66)
- Very low	196	60 (30.6)	0.74 (0.46–1.18)	200	56 (28.0)	1.05 (0.67–1.65)
Extraversion						
- Very high^b^	400	66 (16.5)	1	390	71 (18.2)	1
- High	387	75 (19.4)	1.30 (0.83–2.03)	385	78 (20.3)	0.86 (0.56–1.32)
- Medium	349	80 (22.9)	1.30 (0.82–2.05)	308	65 (21.1)	0.73 (0.47–1.15)
- Low	392	109 (27.8)	1.45 (0.94–2.24)	413	114 (27.6)	1.07 (0.72–1.61)
- Very low	363	123 (33.9)	1.45 (0.94–2.25)	396	122 (30.8)	0.94 (0.62–1.42)
Conscientiousness						
- Very high^b^	426	61 (14.3)	1	454	73 (16.1)	1
- High	297	58 (19.5)	1.51 (0.92–2.48)	263	54 (20.5)	1.21 (0.76–1.93)
- Medium	577	142 (24.6)	1.58 (1.04–2.41)^*^^c^	600	134 (22.3)	1.25 (0.85–1.82)
- Low	310	95 (30.6)	2.02 (1.28–3.20)^**^	283	74 (26.1)	1.13 (0.72–1.76)
- Very low	280	97 (34.6)	1.62 (1.02–2.58)^*^^c^	293	116 (39.6)	1.81 (1.19–2.76)^**^
Openness						
- Very high^b^	292	70 (24.0)	1	304	73 (24.0)	1
- High	347	68 (19.6)	0.67 (0.41–1.09)	335	70 (20.9)	0.73 (0.46–1.15)
- Medium	461	111 (24.1)	0.92 (0.59–1.45)	454	98 (21.6)	0.82 (0.54–1.25)
- Low	409	97 (23.7)	0.75 (0.47–1.20)	402	89 (22.1)	0.76 (0.49–1.18)
- Very low	385	109 (28.3)	0.80 (0.50–1.29)	396	120 (30.3)	1.01 (0.66–1.56)

aOR = Odds ratio adjusted for age, education level, marital status, primary occupation, ethnicity, anxiety and depression symptoms and lack of emotional support assessed in March of the same year (T1 or T4). 95% CI = 95% confidence interval. All results are based on the weighted sample and due to the weighting numbers (*N*, *n*) may differ slightly.

^a^*p*-values before the Benjamini-Hochberg correction (^*^p < .05, ^**^p < .01, ^***^p < .001).

^b^Reference category. Personality factors were examined in May of the same year (T2 or T5).

^c^Not significant after the Benjamini-Hochberg correction.

The cross-wise MLRA showed no significant differences in the prevalence of ADS between females with a certain level of a personality factor before the pandemic compared to females with the same level of the same personality factor during the pandemic. All 50 comparisons were not significant on a *p* < .050 level (see Appendix 3).

### Prevalence of Persistent ADS Among Males

Of the males with pre-existing ADS (at T1/T4), 64.3% and 65.3% also had ADS at T3 and T6 respectively. Table 5 shows that in both periods those with low and very low levels of emotional stability were much more at risk for persistent ADS than those with high/very high levels of emotional stability. Among males with pre-existing moderate-severe ADS and medium emotional stability levels, those during the pandemic were more at risk for persistent ADS in contrast to those before the pandemic. Among males with pre-existing ADS those with very low to high levels of agreeableness, conscientiousness and openness were not more (or less) at risk for persistent ADS (recovered less or more) than those with (very) high levels of these for personality factors, taken the Benjamini-Hochberg corrections into account. Both before and during the pandemic, males with low levels of extraversion more often suffered from persistent ADS than males with very high levels of extraversion.

**Table 5.** Prospective Associations Between Personality Factors and Persistent ADS Among Males With Pre-Existing ADS.

	Persistent Moderate-Severe Anxiety and Depression Symptoms (ADS)					
	Males					
	November 2019			November 2020		
	*N* ^*PA*^	*n (%)*	*aOR (95%CI)* a	*N* ^*PA*^	*n (%)*	*aOR (95%CI)* a
Emotional stability						
- (v.) high^b^	42	10 (23.8)	1	52	10 (19.2)	1
- Medium	44	20 (45.5)	1.90 (0.69–5.25)	56	29 (51.8)	4.53 (1.90–10.77)^***^
- Low	105	73 (69.5)	6.67 (2.72–16.31)^***^	138	87 (63.0)	7.25 (3.34–15.73)^***^
- Very low	170	130 (76.5)	8.35 (3.46–20.19)^***^	155	118 (76.1)	13.49 (6.15–29.58)^***^
Agreeableness						
- Very high^b^	47	28 (59.6)	1	36	20 (55.6)	1
- High	42	22 (52.4)	1.29 (0.50–3.37)	49	27 (55.1)	0.93 (0.38–2.31)
- Medium	52	28 (53.8)	1.29 (0.51–3.27)	55	32 (58.2)	1.36 (0.56–3.34)
- Low	84	55 (65.5)	2.23 (0.95–5.23)	95	51 (53.7)	1.05 (0.46–2.37)
- Very low	137	100 (73.0)	2.73 (1.19–6.26)^*^^c^	166	114 (68.7)	1.69 (0.76–3.73)
Extraversion						
- Very high^b^	33	12 (36.4)	1	48	22 (45.8)	1
- High	50	34 (68.0)	3.26 (1.19–8.95)^*^^c^	61	29 (47.5)	1.23 (0.54–2.79)
- Medium	68	39 (57.4)	2.57 (1.00–6.63)	59	37 (62.7)	2.31 (0.99–5.39)
- Low	94	64 (68.1)	3.88 (1.56–9.68)^**^	91	61 (67.0)	2.80 (1.29–6.06)^**^
- Very low	115	83 (72.2)	3.19 (1.30–7.80)^*^^c^	143	96 (67.1)	2.50 (1.22–5.14)^*^^c^
Conscientiousness						
- Very high^b^	42	17 (40.5)	1	49	25 (51.0)	1
- High	32	19 (59.4)	1.27 (0.45–3.58)	38	20 (52.6)	1.00 (0.40–2.49)
- Medium	83	52 (62.7)	1.46 (0.63–3.39)	81	47 (58.0)	1.33 (0.63–2.82)
- Low	59	36 (61.0)	1.92 (0.79–4.69)	63	33 (52.4)	1.14 (0.51–2.57)
- Very low	145	107 (73.8)	2.55 (1.15–5.69)^*^^c^	172	121 (70.3)	2.18 (1.08–4.42)^*^^c^
Openness						
- Very high^b^	71	45 (63.4)	1	59	37 (62.7)	1
- High	54	26 (48.1)	0.53 (0.23–1.22)	65	37 (56.9)	0.77 (0.35–1.67)
- Medium	67	42 (62.7)	1.46 (0.67–3.17)	78	45 (57.7)	0.82 (0.39–1.72)
- Low	81	54 (66.7)	1.24 (0.57–2.68)	100	68 (68.0)	1.32 (0.64–2.74)
- Very low	88	66 (75.0)	1.74 (0.77–3.91)	102	59 (57.8)	0.74 (0.36–1.56)

(v.) high = high or very high levels.

N^PA^ = N with pre-existing ADS. aOR = Odds ratio adjusted for age, education level, marital status, primary occupation, ethnicity, anxiety and depression symptoms and lack of emotional support assessed in March of the same year (T1 or T4). 95% CI = 95% confidence interval. All results are based on the weighted sample and due to the weighting numbers (*N*, *n*) may differ slightly.

^a^*p*-values before the Benjamini-Hochberg correction (^*^p < .05, ^**^p < .01, ^***^p < .001).

^b^Reference category. Personality factors were examined in May of the same year (T2 or T5).

^c^Not significant after the Benjamini-Hochberg correction.

Due to the lower numbers of males with pre-existing ADS (see Table 5), we limited the cross-wise comparisons of the prevalence of persistent ADS of a certain level of a personality factor during the pandemic, with the prevalence of persistent ADS among males with the same level of the same personality factor before the pandemic, to subgroups with at least 80 respondents with pre-existing ADS (resulting in 20 instead of 50 comparisons). No significant differences were found (see Appendix 4).

### Prevalence of Persistent ADS Among Females

Of the females with pre-existing ADS, 60.9% and 60.5% also had ADS at T3 and T6 respectively. According to Table 6, among females with pre-existing ADS, those with very low levels of emotional stability were more at risk for persistent ADS than those with (very) high levels of emotional stability, both during and before the pandemic. No other differences were found after the Benjamin-Hochberg correction.

**Table 6.** Prospective Associations Between Personality Factors and Persistent ADS Among Females With Pre-Existing ADS.

	Persistent Moderate-Severe Anxiety and Depression Symptoms (ADS)					
	November 2019			November 2020		
	*N* ^*PA*^	*n (%)*	*aOR (95%CI)_a_*	*N* ^*PA*^	*n (%)*	*aOR (95%CI)_a_*
Emotional stability						
- (v.) high^b^	35	11 (31.4)	1	44	10 (22.7)	1
- Medium	54	18 (33.3)	1.07 (0.41–2.80)	57	22 (38.6)	2.14 (0.87–5.24)
- Low	129	78 (60.5)	3.11 (1.32–7.35)^**^^c^	118	57 (48.3)	3.16 (1.41–7.08)^**^^c^
- Very low	255	202 (79.2)	7.13 (3.05–16.64)^***^	256	199 (77.7)	11.77 (5.39–25.69)^***^
Agreeableness						
- Very high^b^	114	75 (65.8)	1	114	66 (57.9)	1
- High	94	59 (62.8)	0.96 (0.52–1.75)	128	75 (58.6)	1.10 (0.63–1.94)
- Medium	85	51 (60.0)	0.97 (0.52–1.81)	70	39 (55.7)	0.87 (0.45–1.68)
- Low	101	74 (73.3)	1.40 (0.75–2.63)	97	63 (64.9)	1.13 (0.60–2.10)
- Very low	78	50 (64.1)	1.08 (0.56–2.07)	67	45 (67.2)	1.65 (0.83–3.28)
Extraversion						
- Very high^b^	75	39 (52.0)	1	57	35 (61.4)	1
- High	68	39 (57.4)	1.20 (0.59–2.44)	87	53 (60.9)	1.15 (0.55–2.39)
- Medium	80	55 (68.8)	1.89 (0.94–3.80)	83	39 (47.0)	0.58 (0.28–1.20)
- Low	122	85 (69.7)	2.07 (1.09–3.93)^*^^c^	125	78 (62.4)	1.02 (0.51–2.02)
- Very low	127	91 (71.7)	1.80 (0.95–3.44)	123	83 (67.5)	0.98 (0.48–1.98)
Conscientiousness						
- Very high^b^	70	39 (55.7)	1	73	41 (56.2)	1
- High	53	33 (62.3)	1.52 (0.70–3.30)	55	28 (50.9)	0.91 (0.42–1.98)
- Medium	146	97 (66.4)	1.80 (0.96–3.37)	140	81 (57.9)	1.29 (0.69–2.44)
- Low	91	62 (68.1)	1.95 (0.97–3.89)	89	50 (56.2)	1.11 (0.56–2.20)
- Very low	112	77 (68.8)	1.88 (0.96–3.70)	117	87 (74.4)	2.48 (1.25–4.91)^**^^c^
Openness						
- Very high^b^	61	39 (63.9)	1	71	41 (57.7)	1
- High	72	42 (58.3)	0.89 (0.42–1.90)	80	45 (56.3)	0.88 (0.43–1.79)
- Medium	104	73 (70.2)	1.36 (0.66–2.81)	103	62 (60.2)	1.10 (0.56–2.13)
- Low	109	70 (64.2)	1.12 (0.55–2.31)	98	55 (56.1)	1.03 (0.52–2.04)
- Very low	125	84 (67.2)	1.15 (0.56–2.39)	123	85 (69.1)	1.76 (0.89–3.49)

(v.) high = high or very high levels.

N^PA^ = N with pre-existing ADS. aOR = Odds ratio adjusted for age, education level, marital status, primary occupation, ethnicity, anxiety and depression symptoms and lack of emotional support assessed in March of the same year (T1 or T4). 95% CI = 95% confidence interval. All results are based on the weighted sample and due to the weighting numbers (*N*, *n*) may differ slightly.

^a^Reference category. Personality factors were examined in May of the same year (T2 or T5).

^b^*p*-values before the Benjamini-Hochberg correction (^*^p < .05, ^**^p < .01, ^***^p < .001).

^c^Not significant after the Benjamini-Hochberg correction.

When limiting the comparisons between subgroups of females with pre-existing moderate-severe ADS to subgroups with at least 80 females (see Table 6) with pre-existing ADS, 28 instead of 50 comparisons were carried out. None of the cross-wise comparisons were significant after the Benjamini-Hochberg corrections (see Appendix 5).

The results showed that for some personality factors lower levels were associated with a higher risk of (persistent) ADS among males, females or both, and for some personality factors not. To additionally test the robustness of these results and especially the non-significant outcomes, we re-analyzed the data in a similar way but by entering continuous scores in the analyses instead of the categorized levels of the personality factors. Results showed that those personality factors for which lower levels were not significantly associated with a higher risk of moderate-severe ADS (see Tables 3 and 4), were also not significantly associated with moderate-severe ADS when those personality factors were treated as continuous scales in the MLRA. In addition, those personality factors for which lower levels were not significantly associated with higher risks of persistent ADS (see Tables 5 and 6), were also not significantly associated with persistent ADS when those personality factors were treated as continuous scales in the MLRA. For the differences in the relationships between personality factors and (persistent) moderate-severe ADS during the pandemic compared to before the pandemic we refer to the results of the cross-wise comparisons within each level of each personality factor, because the aOR’s of the MLRA using the personality factor scores cannot (as described) be compared.

## Discussion

The general aim of the present prospective population-based study was to examine the extent to which the associations between different levels of the Big Five personality factors on the one hand and (persistent) moderate-severe anxiety and depression symptoms (ADS) on the other hand during the pandemic, differed from these associations before the pandemic among males and females. Because of the multiple testing we applied the Benjamini-Hochberg correction.

With respect to the main research question the conclusion can be drawn, in line with Anglim and Horwood (2021), that the associations between the levels of personality factors and the prevalence of moderate-severe ADS during the pandemic compared to these associations in the year before the pandemic were rather stable among both males and females, although some aORs were significant in one study period but not in the other period. For instance, before the pandemic men with low and very low extraversion levels were significantly more at risk for moderate-severe ADS than men with very high levels of extraversion in contrast to during the pandemic. We might speculate that these differences could be explained by advantages in coping with stressors for individuals with greater ease in social situations being counteracted by the social constraints during the pandemic. However, the results of the cross-wise comparisons did not reveal significant differences in the prevalence of ASD between males with low and very low extraversion levels before and males with low and very low extraversion levels during the pandemic.

In line with previous studies, the prevalence of moderate-severe ADS was much higher among those with lower levels of emotional stability than among those with very high levels of emotional stability for both males and females (cf. Kekäläinen et al., 2021; Muro et al., 2021; Proto & Zhang, 2021; Rettew et al., 2021; Rius Ottenheim et al., 2020; Staneva et al., 2022; Szenczy & Nelson, 2021). However, the outcomes of this study show that – when the time between measuring personality and mental health variables (in this case ADS) is kept constant – males and females with lower levels of emotional stability during the pandemic are not more (or less) at risk than males and females with low levels before the pandemic.

Importantly, openness and agreeableness were not independently associated with moderate-severe ADS among males and females, in line with Mansueto et al. (2022) who used the C-19ASS but in contrast to Staneva et al. (2022) and Proto and Zhang (2021) who used the GHQ-12. This finding cannot be attributed to the absence of large differences in raw scores between the five distinguished categories of scores because the Cohens D’s indicated (very) strong differences, but it is unclear which factors can explain these differences in results. Perhaps the (intense) political and societal tensions caused by the Brexit (final agreements were realized at the end of 2020, i.e., during the pandemic), that were absent in the Netherlands, did play a role in the study of Staneva et al. (2022) and Proto and Zhang (2021). In addition, in the analyses we controlled for existing ADS that may help explain the differences.

Findings furthermore showed that both before and during the pandemic males with pre-existing ADS with low and very low levels of emotional stability had considerably more often persistent ADS compared to males with very high levels (70% and 77% vs. 24%, 63% and 76% vs. 19%, respectively). Again, no indications were found that males with pre-existing ADS and a certain level of a personality factor during the pandemic had a higher or lower chance to recover than males with pre-existing ADS and the same level of the same personality factor before the pandemic. Similar patterns were observed among females. The only exception was that among males those with medium emotional stability during the pandemic were more at risk of persistent ADS than those with (very) high emotional stability, in contrast to before the pandemic. Due to cell counts (*n* < 80) we did not perform the cross-wise comparisons to statistically test this finding. We therefore cannot rule out the possibility that medium emotional stability during the pandemic is indeed more often associated with persistent ADS than before the pandemic. We are not aware of corona-related studies using similar study-designs to compare our findings regarding the recovery of ADS with.

It was outside the aim of the present study to examine differences between males and females or if gender moderates the relationship between both although the results among males were often but not always similar to the results among females (cf. Goodwin & Gotlib, 2004; Wang et al., 2023; Zhao et al., 2022). However, with respect to the assessed relationships between personality and (persistent) ADS during compared to before the pandemic, no differences within the subgroup of males nor within the subgroup of females were observed. Future studies are therefore warranted to test the hypothesis that gender does not moderate the relationship between the Big Five personality traits and mental health during (ongoing) stressful circumstances such as the COVID-19 pandemic, in a different way than during circumstances where such stressful events are absent.

### Limitations and Strengths

In this study, we focused on the Big Five personality factors and not on personality disorders or other related factors such as resilience (Truhan et al., 2022). The assessed personality factors are based on self-reports and were examined by the IPIP. Future research is required to examine the extent to which ratings of these factors by others (family, friends, colleagues) or the use of alternative scales yield other results (Anglim et al., 2020; Connelly & Ones, 2010; Pace & Brannick, 2010). All analyses were conducted among male and females, but it was outside the aim of the present study to examine differences between males and females (Lippa, 2010). We did not examine the relationships between the Big Five and moderate-severe ADS within different age-groups (Roberts et al., 2006). We controlled for marital status, but did not examine relationship satisfaction, while personality factors are associated with satisfaction (Malouff et al., 2010). No other relevant mental health problems such as burnout (Alarcon et al., 2009), eating disorders (Cassin & von Ranson, 2005) and risky drinking (Oksanen et al., 2021) were assessed. We have no data on mental disorders assessed with clinical interviews, that would have enriched our study (cf. Hyland & Shevlin, 2024). It was outside the aim of the present study to examine facets of personality factors (Lyon et al., 2021a, 2021b). We have no data to examine if the predictive values of the Big Five are higher at a shorter time interval than 6 months. We were not able to examine the role of these personality factors for mental health problems during childhood and adolescence, that may have increased the risk for mental health problems during adulthood.

However, to the best of our knowledge, this is the first prospective study based on a large traditional probability sample of the general population assessing the predictive values of personality factors assessed before and assessed during the pandemic for mental health before and during the pandemic respectively, while taking relevant confounders such as pre-existing mental health problems into account. To test the robustness or our findings, we repeated the analyses using the continuous scores in the analyses instead of the categorized levels of the personality factors. Findings do not reveal significant associations between personality scores and (persistent) ADS that were not observed in the analyses using the categorized scores.

### Practical Relevance

Insight into possible changes in the predictive values of the Big Five personality factors during this pandemic compared to before the outbreak, is of relevance for targeting interventions to support people who are at increased risk to develop ADS during the present or future pandemic(s). For general practitioners and mental health care professionals, employers and managers, teachers and educators, it is important to know if pre-pandemic knowledge about the influence of personality factors on ADS-levels is applicable to the pandemic. In other words: if their patients, employees, and students exhibited, for instance, high levels of neuroticism, should they be as attentive during the pandemic as “normal” (when the predictive value is more or less similar), much more attentive (when the predictive value increases substantially), or just less attentive (when the predictive value decreases substantially)? Our findings do not provide any indication that personality factors play a profoundly different role in the development of anxiety and depression symptomatology during the COVID-19 pandemic compared to before the outbreak (cf. Anglim & Horwood, 2021). Nevertheless, especially adults with low and very low levels of emotional stability are at high risk for ADS and persistent ADS six months later both before and during the pandemic.

## Supplemental Material

Supplemental Material - The Influence of the Big Five Personality Factors on Mental Health Before and During the COVID-19 Pandemic: A Prospective Study Supplemental Material for The Influence of the Big Five Personality Factors on Mental Health Before and During the COVID-19 Pandemic: A Prospective Study by Peter G. van der Velden, Lutz Wittmann, Carlo Contino, Erik van der Meulen, Marcel Das, and Hendri Adriaens in Psychological Reports

## Data Availability

For the present study data were extracted from the Longitudinal Internet studies for the Social Sciences (LISS) panel. All data, obtained since the first survey in 2007, are available to any interested researcher through the LISS Data Archive, free of charge after registration (open access). See: https://www.dataarchive.lissdata.nl (in English). After registration, the data of six surveys used in the present study can be found at: T1 data: https://doi.org/10.17026/dans-zws-ffz7; T2 data: https://doi.org/10.17026/dans-zmj-5egt; T3 data: https://doi.org/10.17026/dans-zz3-npvp; T4 data: https://doi.org/10.17026/dans-27v-paa3; T5 data: https://doi.org/10.17026/dans-z2c-fzcd; T6 data: https://doi.org/10.17026/dans-xcj-3g8w.

## References

[bibr1-00332941241300949] Aben I. Denollet J. Lousberg R. Verhey F. Wojciechowski F. Honig A. (2002). Personality and vulnerability to depression in stroke patients: A 1-year prospective follow-up study. Stroke, 33(10), 2391–2395. 10.1161/01.str.0000029826.41672.2e 12364726

[bibr2-00332941241300949] Alarcon G. Eschleman K. J. Bowling N. A. (2009). Relationships between personality variables and burnout: A meta-analysis. Work & Stress, 23(3), 244–263. 10.1080/02678370903282600

[bibr3-00332941241300949] Anglim J. Horwood S. (2021). Effect of the COVID-19 pandemic and big five personality on subjective and psychological well-being. Social Psychological and Personality Science, 12(8), 1527–1537. 10.1177/1948550620983047

[bibr4-00332941241300949] Anglim J. Horwood S. Smillie L. D. Marrero R. J. Wood J. K. (2020). Predicting psychological and subjective well-being from personality: A meta-analysis. Psychological Bulletin, 146(4), 279–323. 10.1037/bul0000226 31944795

[bibr5-00332941241300949] Benjamini Y. Hochberg Y. (1995). Controlling the false discovery rate: A practical and powerful approach to multiple testing. Journal of the Royal Statistical Society: Series B, 57(1), 289–300. 10.1111/j.2517-6161.1995.tb02031.x

[bibr6-00332941241300949] Besser A. Priel B. Flett G. L. Wiznitzer A. (2007). Linear and nonlinear models of vulnerability to depression: Personality and postpartum depression in a high risk population. Individual Differences Research, 5(1), 1–29.

[bibr7-00332941241300949] Birkeland M. S. Nielsen M. B. Hansen M. B. Knardahl S. Heir T. (2017). Like a bridge over troubled water? A longitudinal study of general social support, colleague support, and leader support as recovery factors after a traumatic event. European Journal of Psychotraumatology, 8(1), 1302692. 10.1080/20008198.2017.1302692 28451070 PMC5399997

[bibr8-00332941241300949] Bleidorn W. Hopwood C. J. Lucas R. E. (2018). Life events and personality trait change. Journal of Personality, 86(1), 83–96. 10.1111/jopy.12286 27716921

[bibr9-00332941241300949] Bridges K. R. Sanderman R. van Sonderen E. (2002). An English language version of the social support list: Preliminary reliability. Psychological Reports, 90(3 Pt 1), 1055–1058. 10.2466/pr0.2002.90.3.1055 12090497

[bibr10-00332941241300949] Bucher M. A. Suzuki T. Samuel D. B. (2019). A meta-analytic review of personality traits and their associations with mental health treatment outcomes. Clinical Psychology Review, 70, 51–63. 10.1016/j.cpr.2019.04.002 30981042

[bibr11-00332941241300949] Cassin S. E. von Ranson K. M. (2005). Personality and eating disorders: A decade in review. Clinical Psychology Review, 25(7), 895–916. 10.1016/j.cpr.2005.04.012 16099563

[bibr12-00332941241300949] Cohen S. Wills T. A. (1985). Stress, social support, and the buffering hypothesis. Psychological Bulletin, 98(2), 310–357. 10.1037/0033-2909.98.2.310 3901065

[bibr13-00332941241300949] Connelly B. S. Ones D. S. (2010). An other perspective on personality: meta-analytic integration of observers’ accuracy and predictive validity. Psychological Bulletin, 136(6), 1092–1122. 10.1037/a0021212 21038940

[bibr14-00332941241300949] Costa P. T. Terracciano A. McCrae R. R. (2001). Gender differences in personality traits across cultures: Robust and surprising findings. Journal of Personality and Social Psychology, 81(2), 322–331. 10.1037/0022-3514.81.2.322 11519935

[bibr15-00332941241300949] Debast I. van Alphen S. P. J. B. Rossi G. Tummers J. H. A. Bolwerk N. Derksen J. J. L. Rosowsky E. (2014). Personality traits and personality disorders in late middle and old age: Do they remain stable? A literature review. Clinical Gerontologist, 37(3), 253–271. 10.1080/07317115.2014.885917

[bibr16-00332941241300949] Farstad S. M. McGeown L. M. von Ranson K. M. (2016). Eating disorders and personality, 2004–2016: A systematic review and meta-analysis. Clinical Psychology Review, 46, 91–105. 10.1016/j.cpr.2016.04.005

[bibr17-00332941241300949] Feingold A. (1994). Gender differences in personality: A meta-analysis. Psychological Bulletin, 116(3), 429–456. 10.1037/0033-2909.116.3.429 7809307

[bibr18-00332941241300949] Goldberg L. R. (1992). The development of markers for the big-five factor structure. Psychological Assessment, 4(1), 26–42. 10.1037/1040-3590.4.1.26

[bibr19-00332941241300949] Goodwin R. D. Gotlib I. H. (2004). Gender differences in depression: The role of personality factors. Psychiatry Research, 126(2), 135–142. 10.1016/j.psychres.2003.12.024 15123392

[bibr21-00332941241300949] Guan N. Guariglia A. Moore P. Xu F. Al-Janabi H. (2022). Financial stress and depression in adults: A systematic review. PLoS One, 17(2), Article e0264041. 10.1371/journal.pone.0264041 35192652 PMC8863240

[bibr22-00332941241300949] Hakulinen C. Elovainio M. Pulkki-Råback L. Virtanen M. Kivimäki M. Jokela M. (2015). Personality and depressive symptoms: Individual participant meta-analysis of 10 cohort studies. Depression and Anxiety, 32(7), 461–470. 10.1002/da.22376 26014798 PMC4605994

[bibr23-00332941241300949] Hyland P. Shevlin M. (2024). Clinician-administered interviews should not be considered the ‘gold standard’ method of assessing psychological distress. New Ideas in Psychology, 73, 101072–101073. 10.1016/j.newideapsych.2023.101072

[bibr24-00332941241300949] Jourdy R. Petot J.-M. (2017). Relationships between personality traits and depression in the light of the “Big Five” and their different facets, L’Évolution Psychiatrique, 82(4), e27–e37. 10.1016/j.evopsy.2017.08.002

[bibr25-00332941241300949] Karsten J. Penninx B. W. Riese H. Ormel J. Nolen W. A. Hartman C. A. (2012). The state effect of depressive and anxiety disorders on big five personality traits. Journal of Psychiatric Research, 46(5), 644–650. 10.1016/j.jpsychires.2012.01.024 22349302

[bibr26-00332941241300949] Kekäläinen T. Hietavala E. M. Hakamäki M. Sipilä S. Laakkonen E. K. Kokko K. (2021). Personality traits and changes in health behaviors and depressive symptoms during the COVID-19 pandemic: A longitudinal analysis from pre-pandemic to onset and end of the initial emergency conditions in Finland. International Journal of Environmental Research and Public Health, 18(15), 7732. 10.3390/ijerph18157732 34360025 PMC8345535

[bibr27-00332941241300949] Khazanov G. K. Ruscio A. M. (2016). Is low positive emotionality a specific risk factor for depression? A meta-analysis of longitudinal studies. Psychological Bulletin, 142(9), 991–1015. 10.1037/bul0000059 27416140 PMC5110375

[bibr28-00332941241300949] Klein D. N. Kotov R. Bufferd S. J. (2011). Personality and depression: Explanatory models and review of the evidence. Annual Review of Clinical Psychology, 7, 269–295. 10.1146/annurev-clinpsy-032210-104540 PMC3518491 21166535

[bibr29-00332941241300949] Koenen K. C. Ratanatharathorn A. Ng L. McLaughlin K. A. Bromet E. J. Stein D. J. Karam E. G. Meron Ruscio A. Benjet C. Scott K. Atwoli L. Petukhova M. Lim C. C. W. Aguilar-Gaxiola S. Al-Hamzawi A. Alonso J. Bunting B. Ciutan M. de Girolamo G. Kessler R. C. (2017). Posttraumatic stress disorder in the World mental health surveys. Psychological Medicine, 47(13), 2260–2274. 10.1017/S0033291717000708 28385165 PMC6034513

[bibr30-00332941241300949] Kotov R. Gamez W. Schmidt F. Watson D. (2010). Linking “big” personality traits to anxiety, depressive, and substance use disorders: A meta-analysis. Psychological Bulletin, 136(5), 768–821. 10.1037/a0020327 20804236

[bibr31-00332941241300949] Lippa R. A. (2010). Sex differences in personality traits and gender-related occupational preferences across 53 nations: Testing evolutionary and social-environmental theories. Archives of Sexual Behavior, 39(3), 619–636. 10.1007/s10508-008-9380-7 18712468

[bibr32-00332941241300949] Lyon K. A. Elliott R. Ware K. Juhasz G. Brown L. (2021a). Corrigendum to 'associations between facets and aspects of big five personality and affective disorders: Systematic review and best evidence synthesis’ [journal of affective disordersJournal of Affective Disorders volume 288294 (2021), pages 175-188]. 10.1016/j.jad.2021.07.003 34280786

[bibr33-00332941241300949] Lyon K. A. Elliott R. Ware K. Juhasz G. Brown L. J. E. (2021b). Associations between facets and aspects of big five personality and affective disorders: A systematic review and best evidence synthesis. Journal of Affective Disorders, 288, 75–188. 10.1016/j.jad.2021.03.061 33901698

[bibr34-00332941241300949] Malouff J. M. Thorsteinsson E. B. Schutte N. S. Bhullar N. Rooke S. E. (2010). The five-factor model of personality and relationship satisfaction of intimate partners: A meta-analysis. Journal of Research in Personality, 44(1), 124–127. 10.1016/j.jrp.2009.09.004

[bibr35-00332941241300949] Mansueto G. Palmieri S. Marino C. Caselli G. Sassaroli S. Ruggiero G. M. Nikčević A. V. Spada M. M. (2022). The Italian COVID-19 anxiety syndrome scale: Investigation of the COVID-19 anxiety syndrome and its association with psychological symptoms in an Italian population. Clinical Psychology & Psychotherapy, 29(6), 1972–1990. 10.1002/cpp.2767 35771682 PMC9350361

[bibr36-00332941241300949] Means-Christensen A. J. Arnau R. C. Tonidandel A. M. Bramson R. Meagher M. W. (2005). An efficient method of identifying major depression and panic disorder in primary care. Journal of Behavioral Medicine, 28(6), 565–572. 10.1007/s10865-005-9023-6 16249822

[bibr37-00332941241300949] Muro A. Feliu-Soler A. Castellà J. (2021). Psychological impact of COVID-19 lockdowns among adult women: The predictive role of individual differences and lockdown duration. Women & Health, 61(7), 668–679. 10.1080/03630242.2021.1954133 34284689

[bibr38-00332941241300949] Oksanen A. Oksa R. Savela N. Celuch M. Savolainen I. (2021). Drinking and social media use among workers during COVID-19 pandemic restrictions: Five-wave longitudinal study. Journal of Medical Internet Research, 23(12), Article e33125. 10.2196/33125 34662290 PMC8641700

[bibr39-00332941241300949] Oshio A. Taku K. Hirano M. Saeed G. (2018). Resilience and big five personality traits: A meta-analysis. Personality and Individual Differences, 127, 54–60. 10.1016/j.paid.2018.01.048

[bibr40-00332941241300949] Oxford University . (2022). COVID-19 government response tracker. Oxford University. https://www.bsg.ox.ac.uk/research/research-projects/covid-19-government-response-tracker (Accessed 25 February 2022).

[bibr41-00332941241300949] Pace V. L. Brannick M. T. (2010). How similar are personality scales of the “same” construct? A meta-analytic investigation. Personality and Individual Differences, 49(7), 669–676. 10.1016/j.paid.2010.06.014

[bibr42-00332941241300949] Pereira A. T. Cabaços C. Araújo A. Amaral A. P. Carvalho F. Macedo A. (2022). COVID-19 psychological impact: The role of perfectionism. Personality and Individual Differences, 184(2022), 111160. 10.1016/j.paid.2021.111160 34642518 PMC8496947

[bibr43-00332941241300949] Perenboom R. Oudshoorn K. van Herten L. Hoeymans N. Bijl R. (2000). Bepaling afkappunten en wegingsfactoren voor de MHI-5 en GHQ-12 ten behoeve van de berekening van een levensverwachting in goede geestelijke gezondheid (In Dutch). TNO Preventie en Gezondheid. https://repository.tno.nl//islandora/object/uuid:0d5c9820-fe1c-43c2-88ad-84c28db93ca0

[bibr44-00332941241300949] Pradhan M. Chettri A. Maheshwari S. (2022). Fear of death in the shadow of COVID-19: The mediating role of perceived stress in the relationship between neuroticism and death anxiety. Death Studies, 46(5), 1106–1110. 10.1080/07481187.2020.1833384 33064632

[bibr45-00332941241300949] Proto E. Zhang A. (2021). COVID-19 and mental health of individuals with different personalities. PNAS, 118(37), Article e2109282118. 10.1073/pnas.2109282118 34508007 PMC8449367

[bibr46-00332941241300949] Rettew D. C. McGinnis E. W. Copeland W. Nardone H. Y. Bai Y. Rettew J. Devadenam V. Hudziak J. J. (2021). Personality trait predictors of adjustment during the COVID pandemic among college students. PLoS One, 16(3), Article e0248895. 10.1371/journal.pone.0248895 33730075 PMC7968652

[bibr47-00332941241300949] Rius Ottenheim N. Pan K. Y. Kok A. Jörg F. Eikelenboom M. Horsfall M. Luteijn R. A. van Oppen P. Rhebergen D. Schoevers R. A. Penninx B. Giltay E. J. (2022). Predictors of mental health deterioration from pre- to post-COVID-19 outbreak. BJPsych Open, 8(5), Article e162. 10.1192/bjo.2022.555 36039783 PMC9433714

[bibr48-00332941241300949] Roberts B. W. Walton K. E. Viechtbauer W. (2006). Patterns of mean-level change in personality traits across the life course: A meta-analysis of longitudinal studies. Psychological Bulletin, 132(1), 1–25. 10.1037/0033-2909.132.1.1 16435954

[bibr49-00332941241300949] Robinson E. Sutin A. R. Daly M. Jones A. (2022). A systematic review and meta-analysis of longitudinal cohort studies comparing mental health before versus during the COVID-19 pandemic in 2020. Journal of Affective Disorders, 296, 567–576. 10.1016/j.jad.2021.09.098 34600966 PMC8578001

[bibr51-00332941241300949] Scherpenzeel A. Das M. (2011). True longitudinal and probability based internet panels: Evidence from The Netherlands. In Das M. Ester P. Kaczmirek L. (Eds.), Social and behavioral research and the internet: Advances in applied methods and research strategies (pp. 77–104). Taylor & Francis.

[bibr52-00332941241300949] Schmitt D. P. Realo A. Voracek M. Allik J. (2008). Why can’t a man be more like a woman? Sex differences in big five personality traits across 55 cultures. Journal of Personality and Social Psychology, 94(1), 168–182. 10.1037/0022-3514.94.1.168 18179326

[bibr53-00332941241300949] Shokrkon A. Nicoladis E. (2021). How personality traits of neuroticism and extroversion predict the effects of the COVID-19 on the mental health of Canadians. PLoS One, 16(5), Article e0251097. 10.1371/journal.pone.0251097 34010299 PMC8133466

[bibr54-00332941241300949] Sonderen E. V. (2012). Het meten van sociale steun met de Sociale Steun Lijst -Interacties SSL-I en Sociale Steun Lijst -Discrepanties, SSL-D: een handleiding (2e herziene druk) [The measurement of social support with the social support list - interactions (SSL-I) and discrepancies (SSL-D)]. Research Institute SHARE RUG.

[bibr55-00332941241300949] Specht J. Egloff B. Schmukle S. C. (2011). Stability and change of personality across the life course: The impact of age and major life events on mean-level and rank-order stability of the big five. Journal of Personality and Social Psychology, 101(4), 862–882. 10.1037/a0024950 21859226

[bibr56-00332941241300949] Staneva A. Carmignani F. Rohde N. (2022). Personality, gender, and age resilience to the mental health effects of COVID-19. Social Science & Medicine, 301, 114884. 10.1016/j.socscimed.2022.114884 35344776 PMC8915456

[bibr57-00332941241300949] Steel Z. Marnane C. Iranpour C. Chey T. Jackson J. W. Patel V. Silove D. (2014). The global prevalence of common mental disorders: A systematic review and meta-analysis 1980–2013. International Journal of Epidemiology, 43(2), 476–493. 10.1093/ije/dyu038 24648481 PMC3997379

[bibr58-00332941241300949] Stephan Y. Sutin A. R. Luchetti M. Hognon L. Canada B. Terracciano A. (2020). Personality and self-rated health across eight cohort studies. Social Science & Medicine, 263, 113245. 10.1016/j.socscimed.2020.113245 32810694

[bibr59-00332941241300949] Strickhouser J. E. Zell E. Krizan Z. (2017). Does personality predict health and well-being? A metasynthesis. Health Psychology, 36(8), 797–810. 10.1037/hea0000475 28277701

[bibr60-00332941241300949] Strohmaier S. Pillai M. Weitzer J. Han E. Zenk L. Birmann B. M. Bertau M. Caniglia G. Laubichler M. D. Steiner G. Schernhammer E. S. (2024). The relationship between big five personality traits and depression in the German-speaking D-A-CH region including an investigation of potential moderators and mediators. European Journal of Investigation in Health, Psychology and Education, 14(8), 2157–2174. 10.3390/ejihpe14080144 39194938 PMC11353565

[bibr61-00332941241300949] Sutin A. R. Luchetti M. Aschwanden D. Lee J. H. Sesker A. A. Strickhouser J. E. Stephan Y. Terracciano A. (2020). Change in five-factor model personality traits during the acute phase of the coronavirus pandemic. PLoS One, 15(8), Article e0237056. 10.1371/journal.pone.0237056 32760108 PMC7410194

[bibr62-00332941241300949] Szenczy A. K. Nelson B. D. (2021). Neuroticism and respiratory sinus arrhythmia predict increased internalizing symptoms during the COVID-19 pandemic. Personality and Individual Differences, 182, 111053. 10.1016/j.paid.2021.111053 34177026 PMC8213985

[bibr63-00332941241300949] Truhan T. E. Gianniou F. M. Papageorgiou K. A. (2022). Differences in psychiatric symptoms between the UK and Greece prior to and during COVID-19: The roles of subclinical narcissism and mental toughness. Personality and Individual Differences, 185, 111308. 10.1016/j.paid.2021.111308 34642521 PMC8495151

[bibr64-00332941241300949] Velden P. G. van der Contino C. Das M. Wittmann L. (2023). To what extent do post-traumatic mental health and other problems reflect pre-existing problems? Findings from the prospective comparative population-based VICTIMS-study. International Journal of Social Psychiatry, 69(4), 841–852. 10.1177/00207640221140287 36464851 PMC10248301

[bibr66-00332941241300949] Wang C. Havewala M. Zhu Q. (2022). COVID-19 stressful life events and mental health: Personality and coping styles as moderators. Journal of American College Health, 72(4), 1068–1077. 10.1080/07448481.2022.2066977 35471940

[bibr67-00332941241300949] Wang T. Li Q. Liu H. Shi Q. Yang F. Zhang B. Ahmed F. Jian W. Guo J. (2023). Gender difference in the relationship between personality traits and changes in depressive symptoms before and after the COVID-19 outbreak: A follow-up study among Chinese adults. Journal of Affective Disorders, 326, 49–56. 10.1016/j.jad.2023.01.085 36709830 PMC9877321

[bibr68-00332941241300949] Ware J. E. Jr. Sherbourne C. D. (1992). The MOS 36-item short-form health survey (SF-36). I. Conceptual framework and item selection. Medical Care, 30(6), 473–483. 10.1097/00005650-199206000-00002 1593914

[bibr69-00332941241300949] Yu T. Hu J. (2022). Extraversion and neuroticism on college Freshmen’s depressive symptoms during the COVID-19 pandemic: The mediating role of social support. Frontiers in Psychiatry, 13, 822699. 10.3389/fpsyt.2022.822699 35340897 PMC8942765

[bibr70-00332941241300949] Zager Kocjan G. Kavčič T. Avsec A. (2021). Resilience matters: Explaining the association between personality and psychological functioning during the COVID-19 pandemic. International Journal of Clinical and Health Psychology, 21(1), 100198. 10.1016/j.ijchp.2020.08.002 33363581 PMC7753029

[bibr71-00332941241300949] Zhao H. Shi H. Ren Z. He M. Li X. Li Y. Pu Y. Cui L. Wang S. Zhao J. Liu H. Zhang X. (2022). Gender and age differences in the associations between personality traits and depressive symptoms among Chinese adults: Based on China Family Panel Study. Health & Social Care in the Community, 30(6), e5482–e5494. 10.1111/hsc.13972 35993911

