# Supplementary material for: The Influence of the Big Five Personality Factors on Mental Health Before and During the COVID-19 Pandemic: A Prospective Study
Source: Psychol Rep. 2024 Nov 21;129(5):3749–74. doi: 10.1177/00332941241300949 (PMC13530337; doi:10.1177/00332941241300949)
Supplement: Supplementary file 1 [file sj-pdf-1-prx-10.1177_00332941241300949.pdf]

Appendix 1 Mean scores categories of personality factors

	Males						Females					
	2019			2020			2019			2020		
	N	*Mean*	SD	*N*	*Mean*	SD	*N*	*Mean*	SD	*N*	*Mean*	SD
Emotional stability												
- very high	467	44.2	2.5	522	44.2	2.5	308	43.7	2.5	331	43.7	2.3
- high	478	38.5	1.1	449	38.5	1.1	403	38.4	1.1	384	38.5	1.1
- medium	328	34.7	1.1	320	34.6	1.1	379	34.5	1.1	377	34.6	1.1
- low	293	30.6	1.1	299	30.5	1.0	369	30.4	1.1	362	30.5	1.1
- very low	272	24.4	3.5	248	24.5	3.3	433	24.0	3.6	438	24.1	3.6
Agreeableness												
- very high	211	44.9	1.8	182	45.0	1.9	527	45.4	2.0	541	45.5	2.1
high	315	40.9	0.8	314	40.9	0.8	467	41.0	0.8	490	40.9	0.8
- medium	343	38.5	0.5	336	38.5	0.5	346	38.5	0.5	325	38.6	0.5
- low	468	35.6	1.1	478	35.6	1.1	356	35.7	1.1	336	35.7	1.1
- very low	501	29.3	3.6	528	29.6	3.3	196	30.5	2.3	201	30.7	2.2
Extraversion												
- very high	392	41.1	2.9	366	41.5	3.0	401	41.1	2.9	390	41.3	3.1
- high	346	35.5	1.2	351	35.4	1.2	387	35.4	1.1	385	35.3	1.1
- medium	283	32.0	0.8	324	32.0	0.8	348	32.0	0.8	308	31.9	0.8
- low	398	28.8	1.1	360	28.8	1.1	393	28.7	1.1	413	28.7	1.1
- very low	419	22.3	3.3	436	22.2	3.4	363	22.5	3.2	396	22.5	3.4
Conscientiousness												
- very high	357	44.0	1.9	392	44.1	2.1	426	44.3	2.1	454	44.2	2.1
- high	244	40.4	0.5	237	40.5	0.5	297	40.4	0.5	263	40.5	0.5
- medium	525	37.5	1.1	497	37.6	1.1	577	37.6	1.1	600	37.6	1.1
- low	329	34.1	0.8	316	34.1	0.8	311	34.0	0.8	282	34.2	0.8
- very low	384	29.0	2.8	396	29.1	2.9	281	29.4	2.5	293	29.4	2.6
Openness												
- very high	400	42.6	2.5	389	42.6	2.4	291	42.4	2.5	304	42.4	2.3
- high	363	37.9	0.8	349	37.9	0.8	347	38.0	0.8	334	37.8	0.8
- medium	407	35.0	0.8	431	35.0	0.8	461	35.0	0.8	455	35.0	0.8
- low	357	32.1	0.8	344	32.1	0.8	409	32.1	0.8	402	32.0	0.8
- very low	311	27.9	2.4	325	28.2	2.3	384	28.1	2.2	397	28.0	2.4

All results are based on the weighted sample and due to the weighting, numbers (N, n) may differ slightly.

Appendix 2 Crosswise comparisons ADS among males

	Moderate-severe anxiety and depression symptoms (ADS)									
	A1 versus B2					B1 versus A2				
	N[A1]	*n (%)*	*N*[B2]	*n (%)*	*aOR (95% CI)*[1]	*N*[B1]	*n (%)*	*N*[A2]	*n (%)*	*aOR (95% CI)*[1]
Emotional stability										
- very high	231	2 (0.9)	263	6 (2.3)	4.42 (0.64-30.60)	259	6 (2.3)	236	6 (2.5)	1.24 (0.33-4.59)
- high	236	11 (4.7)	223	11 (4.9)	1.09 (0.43-2.75)	225	10 (4.4)	242	12 (5.0)	1.06 (0.42-2.67)
- medium	166	26 (15.7)	166	27 (16.3)	1.04 (0.55-1.98)	155	22 (14.2)	163	24 (14.7)	1.37 (0.60-3.11)
- low	147	58 (39.5)	148	61 (41.2)	0.84 (0.48-1.47)	151	61 (40.4)	145	59 (40.7)	1.34 (0.74-2.43)
- very low	134	81 (60.4)	124	76 (61.3)	1.51 (0.80-2.88)	124	75 (60.5)	138	82 (59.4)	0.90 (0.51-1.61)
Agreeableness										
- very high	103	20 (19.4)	90	18 (20.0)	2.10 (0.79-5.59)	92	17 (18.5)	108	20 (18.5)	1.05 (0.38-2.87)
- high	156	18 (11.5)	157	19 (12.1)	0.97 (0.37-2.55)	157	19 (12.1)	159	16 (10.1)	1.07 (0.44-2.62)
- medium	169	28 (16.6)	168	23 (13.7)	0.56 (0.25-1.23)	168	24 (14.3)	175	32 (18.3)	0.61 (0.26-1.43)
- low	230	41 (17.8)	240	40 (16.7)	0.81 (0.44-1.49)	238	40 (16.8)	238	45 (18.9)	1.06 (0.59-1.92)
- very low	248	71 (28.6)	267	79 (29.6)	1.13 (0.65-1.96)	262	77 (29.4)	252	72 (28.6)	1.10 (0.67-1.82)
Extraversion										
- very high	195	19 (9.7)	184	22 (12.0)	1.11 (0.51-2.44)	182	21 (11.5)	197	17 (8.6)	0.78 (0.36-1.69)
- high	171	27 (15.8)	175	21 (12.0)	0.50 (0.21-1.22)	176	22 (12.5)	176	29 (16.5)	1.29 (0.58-2.88)
- medium	140	29 (20.7)	162	32 (19.8)	2.32 (1.00-5.42)	163	32 (19.6)	143	31 (21.7)	1.22 (0.59-2.54)
- low	199	46 (23.1)	180	41 (22.8)	0.84 (0.45-1.57)	180	42 (23.3)	199	44 (22.1)	1.01 (0.49-2.09)
- very low	207	60 (29.0)	220	61 (27.7)	0.94 (0.52-1.72)	216	62 (28.7)	212	60 (28.3)	1.08 (0.61-1.93)
Conscientiousness										
- very high	176	18 (10.2)	196	17 (8.7)	0.46 (0.19-1.15)	196	20 (10.2)	180	20 (11.1)	1.12 (0.49-2.56)
- high	119	14 (11.8)	119	15 (12.6)	1.09 (0.38-3.13)	118	16 (13.6)	125	15 (12.0)	0.49 (0.17-1.37)
- medium	261	39 (14.9)	252	40 (15.9)	1.52 (0.79-2.96)	245	38 (15.5)	263	38 (14.4)	0.96 (0.51-1.81)
- low	162	27 (16.7)	160	30 (18.8)	0.97 (0.46-2.08)	156	42 (26.9)	168	44 (26.2)	0.71 (0.35-1.45)
- very low	193	78 (40.4)	202	76 (37.6)	0.75 (0.44-1.25)	194	73 (37.6)	191	81 (42.4)	1.34 (0.77-2.33)
Openness										

- very high	199	35 (17.6)	193	28 (14.5)	1.71 (0.81-3.63)	195	30 (15.4)	201	36 (17.9)	1.90 (0.85-4.27)
- high	179	23 (12.8)	174	31 (17.8)	2.38 (1.09-5.19)[*2]	175	33 (18.9)	183	21 (11.5)	0.47 (0.23-0.95)[*2]
- medium	205	37 (18.0)	217	32 (14.7)	0.55 (0.27-1.10)	214	32 (15.0)	202	39 (19.3)	2.06 (1.01-4.17)[*2]
- low	178	40 (22.5)	176	42 (23.9)	0.78 (0.38-1.60)	168	42 (25.0)	179	41 (22.9)	0.62 (0.32-1.20)
- very low	155	44 (28.4)	163	45 (27.6)	0.89 (0.45-1.74)	162	40 (24.7)	157	47 (29.9)	1.73 (0.87-3.46)

[1] p-values before the Benjamini-Hochberg correction ([*] $p < 0.05$).
[2] Not significant after the Benjamini-Hochberg correction.
aOR= Odds ratio adjusted for age, education level, marital status, primary occupation, ethnicity, anxiety and depression symptoms and lack of emotional support assessed in March of the same year (T1 or T4). 95% CI=95% confidence interval. All results are based on the weighted sample and due to the weighting numbers (N, n) may differ slightly.
A1 = subsample A data 2019, A2 = subsample A data 2020, B1 = subsample B data 2019, and B2 = subsample B data 2020 (subsamples are obtained by stratified random sampling for each personality factor level separately).

Appendix 3 Crosswise comparisons ADS among females

	Moderate-severe anxiety and depression symptoms (ADS)									
	A1 versus B2					B1 versus A2				
	N[A1]	*n (%)*	*N*[B2]	*n (%)*	*aOR (95% CI)*[1]	*N*[B1]	*n (%)*	*N*[A2]	*n (%)*	*aOR (95% CI)*[1]
Emotional stability										
- very high	153	8 (5.2)	166	8 (4.8)	1.09 (0.33-3.61)	165	4 (2.4)	155	7 (4.5)	1.57 (0.40-6.21)
- high	197	10 (5.1)	194	15 (7.7)	1.24 (0.51-3.01)	190	13 (6.8)	206	15 (7.3)	1.26 (0.53-3.01)
- medium	187	25 (13.4)	190	29 (15.3)	1.40 (0.74-2.66)	187	29 (15.5)	192	27 (14.1)	0.94 (0.50-1.76)
- low	181	56 (30.9)	182	52 (28.6)	0.88 (0.53-1.48)	179	61 (34.1)	188	59 (31.4)	1.27 (0.78-2.09)
- very low	219	123 (56.2)	218	124 (56.9)	1.12 (0.70-1.79)	220	125 (56.8)	213	121 (56.8)	0.95 (0.61-1.50)
Agreeableness										
- very high	266	63 (23.7)	273	57 (20.9)	0.97 (0.59-1.61)	268	54 (20.1)	261	61 (23.4)	1.25 (0.74-2.09)
- high	232	47 (20.3)	245	58 (23.7)	1.50 (0.85-2.64)	244	56 (23.0)	235	47 (20.0)	0.86 (0.50-1.47)
- medium	172	34 (19.8)	164	35 (21.3)	1.33 (0.69-2.57)	161	35 (21.7)	175	37 (21.1)	1.03 (0.50-2.13)
- low	177	53 (29.9)	172	50 (29.1)	0.79 (0.45-1.38)	164	48 (29.3)	179	53 (29.6)	0.98 (0.51-1.89)
- very low	94	29 (30.9)	105	30 (28.6)	1.13 (0.48-2.64)	96	27 (28.1)	102	31 (30.4)	0.85 (0.37-1.99)
Extraversion										
- very high	201	34 (16.9)	197	37 (18.8)	1.30 (0.70-2.41)	192	34 (17.7)	199	32 (16.1)	0.77 (0.40-1.47)
- high	190	36 (18.9)	193	39 (20.2)	0.69 (0.35-1.36)	192	39 (20.3)	197	39 (19.8)	0.81 (0.43-1.52)
- medium	171	39 (22.8)	156	33 (21.2)	0.76 (0.40-1.46)	152	32 (21.1)	177	41 (23.2)	1.18 (0.61-2.29)
- low	193	54 (28.0)	207	58 (28.0)	1.03 (0.56-1.88)	206	56 (27.2)	199	55 (27.6)	0.95 (0.54-1.69)
- very low	181	61 (33.7)	196	59 (30.1)	0.89 (0.52-1.55)	200	63 (31.5)	182	61 (33.5)	1.17 (0.65-2.10)
Conscientiousness										
- very high	213	30 (14.1)	226	36 (15.9)	0.90 (0.46-1.76)	229	38 (16.6)	213	31 (14.6)	0.55 (0.28-1.05)
- high	147	29 (19.7)	133	27 (20.3)	0.88 (0.42-1.84)	130	27 (20.8)	151	30 (19.9)	0.81 (0.41-1.62)
- medium	291	71 (24.4)	299	67 (22.4)	0.98 (0.60-1.60)	300	66 (22.0)	286	70 (24.5)	1.02 (0.62-1.68)
- low	156	47 (30.1)	142	37 (26.1)	0.60 (0.32-1.11)	139	36 (25.9)	155	48 (31.0)	1.20 (0.63-2.28)
- very low	137	48 (35.0)	148	59 (39.9)	1.88 (0.96-3.67)	145	56 (38.6)	144	50 (34.7)	0.94 (0.50-1.76)
Openness										

- very high	143	35 (24.5)	153	38 (24.8)	1.03 (0.55-1.94)	151	35 (23.2)	148	35 (23.6)	1.02 (0.51-2.03)
- high	177	35 (19.8)	169	36 (21.3)	1.17 (0.61-2.25)	166	34 (20.5)	170	33 (19.4)	1.06 (0.55-2.04)
- medium	227	54 (23.8)	229	49 (21.4)	0.95 (0.55-1.66)	225	49 (21.8)	233	57 (24.5)	1.20 (0.68-2.12)
- low	202	47 (23.3)	202	43 (21.3)	0.79 (0.42-1.48)	201	46 (22.9)	207	50 (24.2)	0.78 (0.43-1.44)
- very low	192	55 (28.6)	200	61 (30.5)	1.16 (0.66-2.03)	198	60 (30.3)	193	54 (28.0)	0.82 (0.45-1.50)

[1] None of the p-values reached the $p < 0.05$ level. The Benjamini-Hochberg was therefore omitted.
aOR= Odds ratio adjusted for age, education level, marital status, primary occupation, ethnicity, anxiety and depression symptoms and lack of emotional support assessed in March of the same year (T1 or T4). 95% CI=95% confidence interval. All results are based on the weighted sample and due to the weighting numbers (N, n) may differ slightly.
A1 = subsample A data 2019, A2 = subsample A data 2020, B1 = subsample B data 2019, and B2 = subsample B data 2020 (subsamples are obtained by stratified random sampling for each personality factor level separately).

Appendix 4 Crosswise comparisons persistent ADS among males

	Persistent moderate-severe anxiety and depression symptoms (ADS)									
	A1 versus B2					B1 versus A2				
	N[A1]	*n (%)*	*N*[B2]	*n (%)*	*aOR (95% CI)*[1]	*N*[B1]	*n (%)*	*N*[A2]	*n (%)*	*aOR (95% CI)*[1]
Emotional stability										
- low	60	42 (70.0)	63	40 (63.5)	0.71 (0.31-1.62)	76	48 (63.2)	45	31 (68.9)	1.26 (0.52-3.09)
- very low	85	65 (76.5)	70	53 (75.7)	1.45 (0.59-3.55)	86	65 (75.6)	85	65 (76.5)	1.19 (0.54-2.60)
Agreeableness										
- low	42	27 (64.3)	53	29 (54.7)	0.71 (0.26-1.91)	43	23 (53.5)	42	28 (66.7)	1.57 (0.59-4.19)
- very low	68	49 (72.1)	76	52 (68.4)	1.07 (0.43-2.62)	89	62 (69.7)	69	51 (73.9)	1.31 (0.60-2.86)
Extraversion										
- low	43	29 (67.4)	47	31 (66.0)	0.64 (0.22-1.86)	44	42 (95.5)	52	44 (84.6)	0.68 (0.22-2.10)
- very low	57	41 (71.9)	74	49 (66.2)	1.10 (0.44-2.75)	68	46 (67.6)	59	42 (71.2)	0.79 (0.31-2.01)
Conscientiousness										
- medium	43	28 (65.1)	37	22 (59.5)	1.24 (0.40-3.88)	44	24 (54.5)	40	25 (62.5)	1.18 (0.37-3.81)
- very low	62	46 (74.2)	86	61 (70.9)	0.86 (0.40-1.84)	85	59 (69.4)	83	61 (73.5)	0.98 (0.45-2.16)
Openness										
- low	38	25 (65.8)	54	36 (66.7)	1.05 (0.38-2.91)	46	32 (69.6)	43	29 (67.4)	0.87 (0.32-2.35)
- very low	43	32 (74.4)	58	33 (56.9)	0.45 (0.16-1.26)	43	26 (60.5)	43	34 (79.1)	2.03 (0.68-6.05)

[1] None of p-values reached the $p < 0.05$ level. The Benjamini-Hochberg was therefore omitted.
aOR= Odds ratio adjusted for age, education level, marital status, primary occupation, ethnicity, anxiety and depression symptoms and lack of emotional support assessed in March of the same year (T1 or T4). 95% CI=95% confidence interval. All results are based on the weighted sample and due to the weighting numbers (N, n) may differ slightly.
A1 = subsample A data 2019, A2 = subsample A data 2020, B1 = subsample B data 2019, and B2 = subsample B data 2020 (subsamples are obtained by stratified random sampling for each personality factor level separately).

Appendix 5 Crosswise comparisons persistent ADS among females

	Persistent moderate-severe anxiety and depression symptoms (ADS)									
	A1 versus B2					B1 versus A2				
	N^{A1}	*n (%)*	N^{B2}	*n (%)*	*aOR (95% CI)*[1]	N^{B1}	*n (%)*	N^{A2}	*n (%)*	*aOR (95% CI)*[1]
Emotional stability										
- low	57	35 (61.4)	61	29 (47.5)	0.61 (0.28-1.34)	57	28 (49.1)	72	43 (59.7)	1.67 (0.76-3.65)
- very low	123	98 (79.7)	124	95 (76.6)	0.75 (0.38-1.44)	132	104 (78.8)	132	105 (79.5)	0.91 (0.47-1.74)
Agreeableness										
- very high	53	35 (66.0)	64	36 (56.3)	0.83 (0.34-2.01)	49	29 (59.2)	61	40 (65.6)	1.08 (0.42-2.79)
- high	42	26 (61.9)	64	38 (59.4)	1.57 (0.61-4.08)	65	38 (58.5)	52	33 (63.5)	0.85 (0.37-1.96)
- low	52	38 (73.1)	53	33 (62.3)	0.57 (0.22-1.48)	44	30 (68.2)	49	36 (73.5)	2.26 (0.65-7.81)
Extraversion										
- medium	40	28 (70.0)	43	20 (46.5)	0.21 (0.06-0.75)[*2]	40	19 (47.5)	40	27 (67.5)	2.53 (0.86-7.42)
- low	62	43 (69.4)	65	40 (61.5)	0.57 (0.23-1.42)	60	38 (63.3)	59	42 (71.2)	1.75 (0.67-4.58)
- very low	63	46 (73.0)	56	38 (67.9)	0.76 (0.31-1.88)	67	45 (67.2)	64	45 (70.3)	1.11 (0.47-2.62)
Conscientiousness										
- medium	69	46 (66.7)	71	40 (56.3)	0.67 (0.29-1.56)	69	41 (59.4)	77	51 (66.2)	1.32 (0.56-3.11)
- low	51	35 (68.6)	42	24 (57.1)	0.68 (0.26-1.76)	48	27 (56.3)	40	28 (70.0)	1.99 (0.73-5.47)
- very low	53	37 (69.8)	69	51 (73.9)	1.48 (0.61-3.56)	47	36 (76.6)	60	41 (68.3)	0.81 (0.32-2.09)
Openness										
- medium	50	36 (72.0)	52	31 (59.6)	0.48 (0.16-1.38)	51	31 (60.8)	54	37 (68.5)	1.32 (0.51-3.41)
- low	52	33 (63.5)	53	30 (56.6)	0.46 (0.15-1.44)	45	25 (55.6)	55	36 (65.5)	1.48 (0.59-3.72)
- very low	63	41 (65.1)	62	43 (69.4)	1.06 (0.46-2.46)	59	41 (69.5)	63	43 (68.3)	0.81 (0.35-1.84)

[1] p-values before the Benjamini-Hochberg correction: [*] $p < 0.05$.
[2] Not significant after the Benjamini-Hochberg correction.
aOR= Odds ratio adjusted for age, education level, marital status, primary occupation, ethnicity, anxiety and depression symptoms and lack of emotional support assessed in March of the same year (T1 or T4). 95% CI=95% confidence interval. All results are based on the weighted sample and due to the weighting numbers (N, n) may differ slightly.

A1 = subsample A data 2019, A2 = subsample A data 2020, B1 = subsample B data 2019, and B2 = subsample B data 2020 (subsamples are obtained by stratified random sampling for each personality factor level separately).
